# Obstacles and problems of ethical leadership from the perspective of nursing leaders: a qualitative content analysis

**Published:** 2017-02-21

**Authors:** Maasoumeh Barkhordari-Sharifabad, Tahereh Ashktorab, Foroozan Atashzadeh-Shoorideh

**Affiliations:** 1 PhD Student in Nursing, School of Nursing and Midwifery, Shahid Beheshti University of Medical Sciences, Tehran, Iran; Department of Nursing, School of Medical Sciences, Yazd Branch Islamic Azad University, Yazd, Iran.; 2 Professor, Department of Medical-Surgical Nursing, School of Nursing and Midwifery, Shahid Beheshti University of Medical Sciences, Tehran, Iran.; 3 Assistant Professor, Department of Nursing Management, School of Nursing and Midwifery, Shahid Behshti University of Medical Sciences, Tehran, Iran.

**Keywords:** *Ethical leadership*, *Nursing*, *Content analysis*, *Qualitative research*

## Abstract

In the nursing profession, leadership plays a significant role in creating motivation and thus enabling nurses to provide high quality care. Ethics is an essential component of leadership qualifications and the ethical leader can help create an ethical atmosphere, offer ethical guidance, and ensure the occupational satisfaction of personnel through prioritizing moralities. However, some issues prevent the implementation of this type of leadership by nursing leaders. The aim of this study was to identify and describe some problems and obstacles in ethical leadership faced by nursing leaders, and to help them achieve more accurate information and broader perspective in this field.

The present study was conducted using a qualitative approach and content analysis. A total of 14 nursing managers and educators were selected purposefully, and deep and semi-structured interviews were conducted with them. Content analysis was performed using an inductive approach.

Three main categories were obtained after data analysis: ethical, cultural and managerial problems. “Ethical problems” pertain to doubt in ethical actions, ethical conflicts and ethical distress; “cultural problems” include organizational and social culture; and “managerial problems” are connected to organizational and staff-related issues.

Nursing leaders put forth various aspects of the problems associated with ethical leadership in the clinical setting. This style of leadership could be promoted by developing suitable programs and providing clear-cut strategies for removing the current obstacles and correcting the organizational structure. This can lead to ethical improvement in nursing leaders and subsequently the nurses.

## Introduction

Today, health care organizations are subject to rapid and fundamental changes aimed at enhancing the quality of service, patient satisfaction and productivity (1). Parallel with these changes, nurses face cases such as heavy workload, increased patient awareness, various problems related to staff skills, lack of resources, low occupational and life quality, and workplace violence (2). However, there is the expectation that nurses should treat patients in an ethical manner and put ethics first in their professional performance. Across the world, nurses are guided to use professional codes that emphasize their obligation to respect, protect and defend the fundamental rights of the people involved in nursing and health care(3). 

One of the most powerful methods to promote ethics in health care and the nursing practice is to role model ethical performance on the managerial level (4). Nurses in formal leadership positions should promote ethics (5), which means they should implement ethical leadership (6), an approach that has attracted much attention in recent years. This style of leadership involves the development of appropriate normal behavior through personal actions and interpersonal interactions, and also promotion of such behaviors in subordinates through bilateral exchanges and strengthening of decision-making (7). Ethical leaders must strive to model and support ethical performance (4) and at the same time be sensitive to moral issues and enhance nurse's performance by fostering respect for human dignity; thus, they can play an important role in promoting patient safety (8), increase the capacity to discuss and act upon ethics in daily activities (9), and support the ethical competence of nurses (10). 

Some studies in this field have indicated that ethical leadership leads to reduced work leave and increased job satisfaction in nurses through decreasing moral distress and creating an ethical milieu (11). Furthermore, this style of leadership boosts confidence in the leader, organizational commitment, and psychological empowerment among the personnel (12). Moreover, it exerts considerable effects on the staff’s creativity and their energetic feeling (13). Disappointment and lack of confidence, commitment and motivation are among the side effects of leaders’ unethical behavior that influence both patients and organizational efficacy negatively (14). Some studies have demonstrated that the leaders’ supportive behavior and confidence in management are essential for stabilizing nursing values. These behaviors include empowering nurses to express their concerns and worries, and providing recommendations for improving their work environment and nursing care (15 - 17). 

The Islamic Republic of Iran is located in the Middle East and enjoys one of the oldest civilizations of the world. It is a developing country with specific ethical values and a population of more than seventy million people. Islam is the formal religion in this country (18), and a combination of Iranian and Islamic cultures form its identity. The religious discipline and cultural beliefs of the Iranian people have entered the health care system and ethical issues are prominent in the patient care protocols (19, 20). The nursing manpower in Iran is estimated to exceed 150,000, forming a considerable portion of the health care staff. The Iranian health system, like any other developing country, suffers from limited manpower and financial resources inconsistent with health care requirements (20). 

Similar to their peers in many other countries, Iranian nurses are dissatisfied with their jobs due to work pressure and shortage of time and resources that prevent them from proper fulfillment of their duties (21). Furthermore, they have been shown to suffer from inappropriate work environment, lack of support, discrimination, conflict, limited opportunities for development, dissatisfaction with work conditions due to heavy workload and unusual work hours, lack of power, and undesirable social status (22). Government policies have resolved nurses’ concerns to some extent, including workload and nurse-patient professional issues; however, there is still the need for a solution to increase the quality of care and improve patient safety (20, 23).

It seems that these problems are imposed by lack of leadership skills in nursing managers. Studies have revealed that task-oriented behaviors are the dominant style of health care leaders and educational systems in Iran (24). Leadership plays a role in creating a culture of care (25), and leadership ethics and confidence in nursing leaders are important components of a healthy work environment culture (6, 17, 26, 27); therefore, this study aimed to identify the barriers that impede the application of ethical leadership in health care settings in Iran. Thus, the authors decided to conduct a qualitative research in this field from the perspective of formal nursing leaders in order to get more detailed information on the nature of the problems and obstacles in ethical nursing leadership.

## Method

The present study used conventional qualitative content analysis and purposive sampling to investigate the problems and obstacles of ethical leadership in nursing. Conventional content analysis is usually the preferred method in studies that focus on elucidating a phenomenon. This design is suitable when there are limited numbers of existing theories or sporadic literature on the phenomenon under study. In this case, the researchers avoided the application of presupposed categories and managed to distill the categories from the data. Hence, the categories are manifested through deduction (28). 

Seeing that in the qualitative approach, the phenomena must be investigated in their natural context, the hospitals and nursing schools of Tehran, Iran, were selected as the research setting. Among the formal nursing leaders, those with at least 2 years of managerial work experience who wished to participate in the study were chosen. Selection criteria for the educators included experience in teaching ethics, leadership status, and publication of books and articles in the field of leadership and ethics. These individuals possessed deep information and experience related to the subject under study (they were key informants) and could provide the researcher with much information. As decision-makers in the health care system, clinical leaders and university authorities cannot be separated from each other, and the emergence of ideas on ethics requires the participation of both; therefore, nursing educators were also involved in this research. Moreover, the participating educators had some experience in clinical settings and leadership, and a number of them were occupied in the capacity of formal leaders at the time. This study tried to cover a sample with great variety in age, gender, management level, department, and work experience. To find the more experienced participants with a richer reservoir of data, the initial sample was used, which included nursing educators with a 22-year management experience at various levels of nursing as well as teaching ethics. 

A total of 14 individual interviews were conducted. Data saturation was achieved after 11 interviews, but an additional three were carried out to reach certainty**. **As confirmed by research participants, the interviews were held in a quiet room in their workplace at hospitals and colleges.

All interviews were conducted by the first author, a female PhD candidate in nursing, and a nursing instructor. She has received the customary training for PhD students to prepare for doctoral dissertations, and has also completed a content analysis workshop. 

After obtaining the approval and written informed consent from the participants, deep and semi-structured interviews were conducted to collect the data. The interviewer began with general questions and proceeded to ask, “Where is the position of ethics in your leadership?” Then, based on the goals of the study, the more detailed questions followed, for instance: “Have you ever been in a situation where you did not do what was ethical despite being aware of it? Please explain.” and “What happened that led you to behave like that?”

In order to obtain more data and clarify certain issues, some probing questions were also asked, such as: “Can you give us an example? What did you mean by that? Can you explain further?”

Individual interviews lasted for 35 to 90 minutes and the participants were then asked to discuss any remaining issues that came to mind. The interviews continued till data saturation was achieved so that no new code of data could be retrieved. 

In this study, the common points were identified, coded and categorized by using latent content analysis. In this method, the researcher searches for specific concepts and the meaning of all the data within the context, and will then design the structural model that can relate the meaningful classes with similar themes (29). 

All interviews were recorded on tape and the transcripts were typed, reviewed and coded at the end of each interview. To observe the principle of confidentiality, participants’ names were not revealed. Instead, each of them was given a specific number and their important particulars such as age, type of degree, and managerial experience were recorded. 

Data were analyzed simultaneously and continuously by collecting information. Semantic units were extracted in the form of initial codes or open codes from the interviews. The codes were reread several times and placed in subcategories on the basis of similarity and proportion of the participant expressing the same topic. Next, the subcategories were compared with each other and those with similar characteristics were combined to create wider categories, which were presented once more. 

Some of the measures taken to enhance data accuracy included prolonged involvement with the topic, confirmation of findings by the participants, and observer reviews. To ensure dependability of the data, in addition to the members of the research team, two experts out of the research team were asked to evaluate the interviews, codings and categories. As regards the conformability of the data, all research steps, including data collection and analysis, observer reviews and the research process were documented on a regular basis. To enhance transferability, the entire process of the research and all the work done in the course of the study were prepared in clear and accurate written form to enable others to track and study population characteristics.

This study was approved by the Human Research Ethics Committee of Shahid Beheshti University of Medical Sciences in Tehran, Iran. In the course of the study, permission was obtained from the authorities of hospitals, departments and colleges. The interviewer began by introducing herself and explaining the purpose of the research to the participants, who were then asked to complete the demographic questionnaire and informed consent. Moreover, permission to record the interviews and take notes was obtained from the participants, and confidentiality and subjects’ freedom to participate in or withdraw from the research was observed. The participants were also assured that their names will not be revealed under any circumstances. Interview tapes are kept anonymously in a safe place and can be accessed only through codes assigned by the researcher.

## Results

There were 14 participants with a mean age of 46 years and an average management experience of 12 years. In terms of managerial level, 3 were supervisors (all matrons), 6 were head nurses, and 5 were nursing educators (Table 1). 

Analysis of the handwritten notes on participants’ experiences of the problems associated with ethical leadership in nursing resulted in the formation of 73 original codes, which were reduced to 21 after merging similar codes. Eventually, three main categories and 7 subcategories emerged that are presented in Table 2. Major groups included ethical problems, cultural problems and managerial problems.

**Table 1:** Characteristics of the study participants

**Characteristics**		**Number**
Gender	Male	6
	Female	8
Education Level	Bachelor’s degree	6
	Master’s degree	3
	Doctoral degree	5
Position	Head nurse	6
	Supervisor	3
	Nursing educator	5

**Table 2:** Generated categories, subcategories and examples of codes

**Categories**	**Subcategories**	**An Example of the Code**
Ethical Problems	Doubt in ethical act	- Negative outcome of introducing a role model to staff - Uncertainty about how to deal with staff
	Ethical conflict	- Conflict between the needs and expectations of nurses - Conflict in meeting the similar needs of employees - Conflict between leaders’ values and the values of the organization
	Ethical distress	- Discomfort following the implementation of procedures in accordance with organizational policies and rules - Dissatisfaction caused by being forced to perform certain tasks due to shortage of staff
Cultural Problems	Organizational culture	- Absence of a culture of democracy in the organization - Lack of the proper culture in introducing the role model
	Social culture	- Negative public perception of the nursing community - Negative feelings of patients’ families with regard to the night shift - Attitude of the majority of the population to nurses as physician assistants
Managerial Problems	Issues related to organization	- Lack of power and authority in recruitment - Physician-oriented system - Low regard for the nursing profession - Lack of facilities - Characteristics of the clinical environment
	Issues related to staff	- Staff's abuse and bullying - Undesirable behavior such as speaking ill of each other - Understanding of justice among personnel

From the perspective of the nursing leaders in this study, there are three major types of problems and obstacles in ethical leadership: ethical, cultural and managerial problems.

**Ethical problems**

Ethical problems were among the categories abstracted from the data. In nursing leadership, these types of problems are sophisticated and pervasive due to their far-reaching implications and varied solutions on the one hand, and uncertain events and personal impressions on the other. The reason is that acts performed by nurse leaders affect staff, patients and other people. If these acts affect others inconsistently, or harm them in an uncontrolled manner, ethical problems arise. This category consists of three subcategories of doubt in the ethical act, ethical conflict, and ethical distress.

***A) Doubt in the ethical act:*** Quite often the consequences associated with ethical choices are not clear, and this will make leaders doubt their ethical performance. Participant number 2 who has a PhD in nursing and 8.5 years of management and leadership experiences at various levels puts it this way:

*[I introduced an ethical example to others in a meeting, but my ethical act was associated with an adverse result, which made a group of people upset. I thought I was introducing a model, but others thought that I was accusing them of not acting ethically. Now, I do not know if it was ethical or not, if it is ethical, so why didn’t what we read about in books work?] *(Participant No.2)

Some participants stated that doubt in the ethical act is a challenge in nursing leadership. For example, participant number 11, a 45-year-old nurse with 9 years of experiences in different wards says:

*[I try to behave in the correct way and pay attention to these issues, for example, I try to be friendly with the personnel and respect them, but I see they have different perceptions. They take advantage of my kindness…. Sometimes I think* *maybe I am not doing it right* *and I should be like the others and treat them in a way so they won't dare to disobey me.] *(Participant No. 11)

***B) Ethical conflict:*** Differences in ethical values in practice will lead to ethical conflicts. Participants admitted that sometimes they are involved in situations where the needs and expectations of nurses are in conflict with each other. Participant number 6, a 38-year-old nurse with a 6-year experience in head nursing and supervisory says in this regard:

*[I have a novice among my personnel. Well, ethically I should team her up with a more experienced employee, but in such cases, the more experienced ones will complain because this will increase their workload. They may request to work with another person in the same shift. See, you are involved in a situation where you don’t really know what to do, so what does ethics say here?] *(Participant No. 6) 

Another aspect of the conflict is that both employees have similar desires, and therefore the nurse leader experiences negative feelings as to whose needs to meet under the circumstances. Participant number 14, a master of nursing with 20 years of management experiences at various levels (head nurse, supervisor and matron) says:

*[When planning shifts, you can't always be fair; for example, on many occasions, two employees needed a day off on the same date, and it was very important for both of them to take that particular day off. On the other hand, it was impossible for me to let both of them have a day off because I was short on staff, so I had to choose one.] *(Participant No. 14) 

Participants stated that sometimes there is a conflict between their values and beliefs and those of the organization. As nursing directors, they are expected to act in accordance with organizational values and justify them for the personnel, even though they might have different opinions. Participant number 9, a 44-year-old nursing PhD with 5 years of management experiences states:

*[It is interesting that sometimes high-level executives or security managers tell us to be tactful, and by that they mean we should tell a lie, pretend to be more skillful than we actually are, and ignore many things. I'm not like that.] *(Participant No. 9)

Participants believed that ethical leadership is harder and more complex in the clinical setting than other work environments such as nursing education due to a high rate of ethical conflict: 

*[In my opinion, there is a great deal of ethical conflict about leadership and management issues in hospitals…. You don’t know who’s right, the patient or the ward nurse, because nurses work really hard on various shifts and are under a tremendous amount of pressure. On the other hand, you see that patients are also right and want to receive the best service possible.] *(Participant No. 2)

***C) Ethical distress:*** Participants stated that when they are faced with obstacles that force them to act against their ethical beliefs, they feel discomfort, dissatisfaction and frustration. They acknowledged that they are often involved in situations where they know the right way to do things, but organizational policies and rules and lack of support from superiors make it impossible for them to perform their duties appropriately and this causes them distress and discomfort. 

*[Sometimes I'm asked to do something that is not within the rules, but is right by logic, reason and humanity. I know that if I do that, the consequences will come back to me and* *I’ll be held accountable later. These things make me sad and angry.] *(Participant No. 2)

Another condition that causes ethical distress in nursing leadership is lack of adequate and skilled manpower in wards. In this regard, participant number 5, a nursing expert with a 20-year experience of head nursing in different wards says:

*[The ward nurse calls me and says her child is sick and asks me to give her a day off or change her shift. I don’t want to say no to her, but I am short on staff, so I oblige her to come. The situation makes me very sad, but I do not have any other choice.] *(Participant No. 5)

Similarly, participant number 9, who is a 44-year-old PhD with 5 years of management experience, states:

*[I am forced to put someone that I don’t trust in charge. I just do this so that somebody fills the post, because I do not have any efficient workforce. It is clear that I am very dissatisfied with this situation, but there is nothing I can do.] *(Participant No. 9)

**Cultural** **problems**

In data analysis, cultural problems were abstracted as the second category. Like any other institution, hospitals and health care centers have their own beliefs and norms that determine the way of thinking, behavior and performance of employees within the organization. The beliefs and opinions of organization members reflect those of the society in which they grew up. The nursing leaders in this study noted cases that can be classified as cultural problems, including two subcategories of social culture and organizational culture.

***A) Social culture:*** Culture is a model of values, beliefs and attitudes of people in every society, and the culture of any organization, including health care centers, is therefore no exception. Lack of respect for the nursing profession and the negative public image associated with it were among the cases that the participants pointed out. This negative attitude has an impact on the self-confidence and motivation of nurses, including nursing leaders. Leaders with low levels of self-confidence and motivation cannot play their leading and supportive role as may be expected. In this regard, participant number 9 states:

*[When I passed the nursing entrance exam, I did not get much positive feedback. My family got upset that I was going to nursing school, and, well, that's all it takes to diminish one’s self-confidence. It is much better now, but the impact still remains.] *(Participant No. 9)

The Iranian society is a family-centered one, and this creates a negative attitude towards women working late hours and night shifts. Some participants mentioned this as an obstacle to their ethical practice. For example, participant number 13 says in this regard:

*[The night shift is a problem for some female personnel, as their families simply don't understand that nursing means working nights and* *circulating shifts. Very often you see families call and say, “Don’t assign X for the night shift.] *(Participant No. 13)

***B) Organizational culture:*** Organizational culture is a control agent that shapes the attitudes and behavior of employees, and the participants pointed out the problems in this area as one of the cases. For example, participant number 3, a 52-year-old nursing PhD with 7 years of experience in management states:

*[One problem is that there is no culture of democracy in our organizations, that is, when you believe that you should lead a group in a democratic way, you will get hurt.] *(Participant No. 3)

Another participant says:

*[People are encouraged to introduce a model in ethical leadership. Well, I did, and it caused annoyance, which means that doing so will bother people and is* *not conceived as a norm* *for employees.] *(Participant No. 2)

**Managerial problems**

Another issue in the field is connected to managerial problems. These problems revolve mainly around axes such as inappropriate procedures, guidelines and evaluations as well as the poor performance of the nursing staff. This category consists of two subcategories: issues related to the organization and issues related to staff.

***A) Issues related to the organization:*** Lack of authority in recruitment, low regard for nursing, shortage of manpower and resources, and certain clinical characteristics were mentioned by the participants in this respect. Of the above-mentioned factors, they considered the first as one of the most challenging. For example, participant number 13, 44 years old, with 15 years of management experience states:

*[The problem with our job is that the employment criteria are the same for all the staff; the payment is also the same… yet, the work done by different individuals varies from individual to individual. The head nurse does not have the authority for proper employment and payments; this is an obstacle to the administration of justice.] (Participant 13).*

Participants had also experienced a lack of respect for nurses. They said that sometimes decisions are made for the nursing department without considering their opinions. Nurses are simply asked to implement those decisions, and do not even receive explanations and clarifications on the process. For example, participant number 8 says:

*[Apart from the fact that sometimes they make decisions about us without asking for our opinions, they do it without even telling us how to implement those decisions, let alone expect us to act ethically.] *(Participant No. 8)

One source of power for leaders is their authority, which is based on reward and punishment. In order to use these power sources fairly, it is essential to evaluate staff performance, the most important purpose of which should be the improved quality of patient care and safety. But nursing leaders believe that the common evaluation method of staff performance is not fair:

*[We deal with humans in our workplace, so a fair evaluation of personnel is hard. For example, they provide a patient with education, their care quality is different from each other, and it is difficult to evaluate how much harm is brought about due to the errors they make.]* (Participant No. 13)

Characteristics of health care organizations and in particular clinical environments, which are a component of the nursing profession, have created challenges for ethical leadership:

*[Given the circumstances of the clinical environment, it is so hard to establish democracy and justice. There are guidelines, instructions and policies in the clinical environment that might be in conflict with the spirit of democracy.]* (Participant No. 3)

***B) Issues related to staff:*** Ethical or unethical behavior and performance of the nursing staff can trigger positive or negative consequences at the organizational level; thus, non-observance of some ethical standards is a source of concern for efficient leaders. One of the things referred to by the participants in this study was abuse and bullying on the part of nurses. Participant number 10, a specialist nurse with 18 years of management experience states:

*[There are some people who complain no matter how you plan their shifts. You give them good shifts that they like, but when they see that others got good shifts too, it’s like they feel jealous…. Sometimes they disrupt your ward’s atmosphere, for example by irritating someone, and then it is no use trying to be just.]* (Participant No. 10)

Adherence to ethical values on the personal level is the individual dimension of ethics, and a lack thereof will cause greed, selfishness, speaking ill of others, and so on. Such behaviors will lead to an escalation of conflict in the organization. Participant number 9, a 44-year-old PhD with 5 years of management experience says:

*[When I was manager, I noticed that people can perform a lot of unethical acts and sometimes they do whatever they can to cause harm to one another.]* (Participant No. 9)

Participants believed that members of the staff are different from each other, even if placed in similar circumstances. One challenge faced by nurse leaders was lack of acceptance of individual differences among the staff, and a sense of injustice. In this regard, participant number 7, 47 years old, with 9 years of management experience at various levels states:

*[There are too many individual differences among personnel. Well, one considers these in planning shifts and such, but to make them understand these differences is another story. For example, none accepts that one person’s quality of work differs from that of others. They may think that I pay more attention to X and have someone’s back more than others.]* (Participant No. 7)

## Discussion

The findings of the present study were similar to those of other studies on this topic. Three main categories were identified, indicating the participants’ perceptions and experiences of ethical leadership barriers and problems in the sociocultural context of the Iranian health care setting. It should be mentioned that all formal nursing leaders in this study showed a kind of positive feeling and interest with respect to this leadership style. This was to be expected as members of the nursing workforce are committed to ethical practice in their profession (6). 

Ethical problems were among the abstracted categories in this research. Nursing leaders experienced doubt in the ethical act, ethical conflicts and distress in the clinical setting. Other studies with similar findings have also mentioned these issues (30 - 33). 

The results of this study demonstrated that the unexpected outcomes of ethical behavior could cause nursing leaders to hesitate about performing ethical acts. One issue that is raised in emergence of ethical complaints is that not all criteria used to justify ethical beliefs are fair, as they are affected by the ethical beliefs of a culture or a person (34). In one study, Scott states that identification of a right performance is a challenge to organizational resources. She believes that consideration of others’ views, detection of unintended consequences and engaging in continuing education is useful for tackling this challenge (30).

Today, ethical conflicts and controversies are inevitable in health care organizations round the globe, and this may lead to ethical distress (35). The study participants had experienced this conflict between the nurses’ expectations and needs, their own values and beliefs and those of the organization, and the needs of patients and the personnel. Other studies have shown that in the changing health care environment, ethical leaders encounter three different values, i.e., individual (power, value and respect), professional (patient-centered care) and organizational values (competition, risk-taking and position) (31). Lack of balance between care and management duties may lead to ethical conflict in leaders (32). Nursing managers experience conflicts between individual and organizational ethics, especially when they cannot provide quality care due to organizational constraints (33). 

The results of this study showed that obstacles such as organizational policies and rules, lack of support from superiors, and lack of sufficient and qualified manpower will weaken leaders’ capability to perform ethical acts and create ethical distress. These findings are similar to those of the study by Gaudine and Beaton. 

Their findings revealed that disagreement with organizational policies over employee discipline, centralized decisions, and lack of ethical resources available to nurses are among the sources of ethical distress in ethical leaders (36). Shirey and Fisher believe that ethical distress arises from role complexity and increased stress, and is a source of psychological stress associated with conflicts experienced by nursing managers (37). However, due to the descriptive and general nature of problems, sources of ethical distress were not specifically investigated in this study.

Leadership and management are affected by cultural, social, and economical factors. Cultural problems were one of the categories extracted from the participants’ statements. The role of culture in human behavior is one of the most important concepts discussed in behavioral sciences. In the present study, participants considered cultural and professional identity as one of the factors contributing to the promotion of the profession and professional attitude. Positive or negative cultural factors can be seen in any society. For example, negative attitudes towards nurses expressed by the participants can be effective on their self-confidence, authority, professional socialization process and professional identity. These findings are approved by other studies as well (21, 38 - 45). The sociocultural context can greatly affect the leadership course and efficacy factors, as well as the approval of leadership features in a specific social culture (46 - 48). The assessment and interpretation of leaders’ behavior and characteristics are related to various sociocultural backgrounds. 

The participants experienced improper organizational culture such as an absence of model acceptance, which affected their ethical leadership. The cultural values of an organization are usually a reflection of the society and the environment in which it belongs. Some other studies have suggested that the organizational culture is correlated with individual and leadership efficacies. 

The constructive aspects of organizational culture encourage individuals to find a way for self-correction and acquiring job satisfaction (49). Similar to this study, Aitamaa et al. referred to organizational and cultural factors such as lack of respect for, and the general negative attitude towards the nursing profession in health care organizations, stating that this culture has a negative impact on nurses’ work motivation (39). Moreover, research on leadership points out that an understanding of the organizational culture cultivates the efficacy of leadership (50), and that ethical leadership plays the mediating role in the relationship between the organizational culture and personnel consequences such as satisfaction, extra effort, effectiveness (51). 

Data analysis confirmed that managerial problems are obstacles for ethical leadership. Issues related to the organization such as lack of power and authority in recruitment, low regard for the nursing staff, shortage of manpower and resources, and specific clinical features were among the cases referred to by the participants. This is not consistent with the findings by Fradd, who emphasizes the important role of nursing mangers in organizational decision-making in the scope of nursing    (52) . Of course, other studies have highlighted the limited power and influence of nursing managers and nurses and lack of their participation in organizational decision-making and inequality of professions in organizations (21, 39, 42). 

In this study, participants referred to behaviors on the part of the personnel that disrupted the environment and had an impact on the quality of care, thus challenging ethical leadership. For instance, mistreatment, bullying and behavioral disorders such as defamation were among the cases referred to by the participants. 

Behaviors such as bullying are quite common in the nursing profession, as has been reported in many studies    (53 - 57) . In a study by Gilbert et al., 86.2 % of nursing managers witnessed bullying by the nurses and 52% of them were victims of bullying (56). Aitamaa et al. also investigated issues common among the staff, for instance lack of cooperation, help and trust, groundless criticism, and non-commitment to group decisions (39). Latent behaviors such as insulting or humiliating others and backbiting are among destructive overt behaviors (58, 59). The participants mentioned the difference in attitudes towards the same subject as one cause of these behaviors. This is consistent with the findings by Aitamaa et al. For example, in planning personnel shifts and holidays, some see justice as assignment of various shifts in equal numbers, while others believe in planning shifts by taking into account the wants and life conditions of personnel (39). 

## Conclusion

This study showed that despite the emphasis on ethical leadership in existing research, there are some barriers and problems in the implementation of this style of leadership. These obstacles have various aspects in ethical, cultural, and managerial domains. Identification of these factors can promote the ethical dimension of leadership. Health care policy makers may utilize the findings of this study to formulate programs and clear-cut strategies to remove these barriers and improve organizational structure and thus promote this style of leadership. Moreover, development of organizational ethical codes for guiding the performance of nursing leaders in confrontation with these problems may be helpful. Ethical leadership is feasible through correction of social and organizational cultures, and securing the public confidence in nursing from organizational and extra-organizational aspects. In conclusion, nursing leaders are required to consider the individual and occupational features and characteristics of personnel when approaching these problems. 

Discussions about the nursing profession and nursing leaders’ conditions can improve the standards in this regard. The leaders themselves play a key role in such discussions and should make their actions clearer and more specific. The findings of this study may help with the development of an instrument for investigating the barriers and problems of ethical leadership in nursing.

Further studies are required on ethics in management and research, specifically in the case of each of the obstacles, causes of ethical problems, and their frequency and severity as well as their differences in various levels of management. Presently ,there is very limited information about the values, resources and mechanisms to resolve ethical problems, and that could be the basis for future research.
